# Identification of the New Metabolite of Nebivolol Using Liquid Chromatography Coupled with High-Resolution Mass Spectrometry and Chemometrics

**DOI:** 10.3390/molecules27030763

**Published:** 2022-01-24

**Authors:** Jakub Trawiński, Michał Wroński, Maciej Gawlik, Robert Skibiński

**Affiliations:** Department of Medicinal Chemistry, Faculty of Pharmacy, Medical University of Lublin, Jaczewskiego 4, 20-090 Lublin, Poland; jakub.trawinski@umlub.pl (J.T.); wronski.m@o2.pl (M.W.); maciej.gawlik@umlub.pl (M.G.)

**Keywords:** chromatography, mass spectrometry, drug metabolism, biotransformation, HLM, toxicity

## Abstract

In this study, the phase I hepatic metabolism pathway of a cardiovascular drug nebivolol was proposed on the basis of a human liver microsomes assay with the use of LC-HR-MS coupled with the chemometric method. Six biotransformation products were found with the assistance of chemometric analysis. Five of them were identified as the previously reported products of alicyclic hydroxylation and dihydroxylation, aromatic hydroxylation, as well as alicyclic oxidation of the parent compound. Moreover, one metabolite, not reported so far, was found to be a product of N-dealkylation of nebivolol—2-amino-1-(6-fluoro-3,4-dihydro-2*H*-1-benzopyran-2-yl)ethan-1-ol. The novel metabolite was submitted to an in silico toxicity analysis to assess its biological properties. The applied computational methods indicated a significantly elevated risk of its mutagenic activity, compared to the parent molecule. Several metabolites of the nebivolol described in the literature were not detected in this study, indicating their non-hepatic origin.

## 1. Introduction

Drug metabolism studies are helpful in predicting the fate of a drug in the body and, thus, in determining the appropriate drug dosage regimen. Identification and characterization of metabolites of the drug candidate and specific enzymes responsible for its metabolism provides an opportunity to assess the impact of metabolites on the safety and efficacy of the drug. Noteworthy, metabolism of xenobiotics is a complex process that has two major phases. The phase I reactions are decomposition processes including also the introduction of polar groups (e.g., hydroxyl group) into the parent structure. These biotransformation reactions, based on redox processes, may lead to the formation of free radicals, which are highly reactive and might establish irreversible conjunctions with biological tissues, especially dangerous for living organisms (resulting in, e.g., cancerous processes). During the phase II reactions, the intermediates received in phase I undergo connections with the endogenous compounds (e.g., glucuronic acid) and then the excretion process takes a place. It should be noticed that the majority of the metabolic reactions are mainly catalyzed by cytochrome P450 enzymes, and thus this kind of research is often performed with the use of in vitro human liver microsomes (HLM) protocol [1,2,3,4,5,6].

Nebivolol [1-(6-fluoro-3,4-dihydro-2*H*-1-benzopyran-2-yl)-2-{[2-(6-fluoro-3,4-dihydro-2*H*-1-benzopyran-2-yl)-2-hydroxyethyl]amino}ethan-1-ol] is a potent and cardioselective β1-adrenergic antagonist with no intrinsic sympathomimetic properties, and is endowed with the ability to release nitric oxide from the cardiovascular endothelium. It is administered as a racemic mixture of two enantiomers in equal ratios. For the selective β1-adrenoceptor, the D-nebivolol is mostly responsible for the antagonist activity, but for the vasodilatory action, the L-nebivolol isomer is the primary contributor [7].

Nebivolol is subject to extensive first-pass metabolism [8]. The major pathway for the metabolism of this drug is catalyzed by CYP2D6 and to a lesser extent by CYP2C19 and CYP3A4. The main product of this reaction is 4-hydroxynebivolol [9,10]. A study in mouse microsomes showed that at least one substance formed during liver cell metabolism is capable of activating nitric oxide (NO) production in endothelial cells by inducing a β2-adrenergic receptor-mediated rise in endothelial [Ca^2+^]. Nebivolol itself had no such effect. In contrast, in another study both nebivolol and its 4-keto metabolite were found to increase NO bioavailability by antagonizing its catabolism induced by oxidative forms [11].

Additionally, it was reported that quantitative differences between nebivolol metabolic pathways depend on the efficiency of debrisoquine hydroxylation [8,12,13,14]. The most abundant compounds were hydroxylated metabolites in subjects phenotyped as extensive debrisoquine metabolizers. In the poor metabolizers, aromatic hydroxylation was deficient, while the major metabolic pathway appeared to be *O*-glucuronidation of unchanged nebivolol or subsequent alicyclic mono-oxidation [13,14]. In both groups, alicyclic hydroxylation and glucuronidation of the parent drug or hydroxylated metabolites were equally important metabolic pathways, while N-dealkylation was less important [8].

A comprehensive description of nebivolol metabolism was provided by J. Hendricks et al. [15]. In their work, urine samples from different species (human, dog, rat, and rabbit) were studied. According to the given results nebivolol was extensively metabolized in all tested species. In humans, nebivolol undergoes both aromatic hydroxylation and alicyclic mono- and dihydroxylation. As a result, aromatic hydroxy-, alicyclic mono-, and dihydroxy-metabolites are formed. During aromatic hydroxylation, the hydride shift (NIH) occurs, resulting in the migration of the ring substituent and the formation of 7-fluoro-6-hydroxy-nebivolol or 5-fluoro-6-hydroxy-nebivolol. In rabbit, dog, and rat, combined dihydroxy-derivates were also observed. Both alicyclic mono- and dihydroxy-metabolites can be dehydrogenated to 4-ketonebivolol and alicyclic hydroxy-4-ketonebivolol, respectively. Oxidative N-dealkylation of nebivolol results in formation of 1-(6-fluoro-3,4-dihydro-2*H*-1-benzopyran-2-yl)ethane-1,2-diol with subsequent oxidation to (6-fluoro-3,4-dihydro-2*H*-1-benzopyran-2-yl)(hydroxy)acetic acid. Both aromatic hydroxylated and alicyclic oxidized metabolites have pharmacologic activity similar to that of the parent drug, while N-dealkylated and glucuronides are inactive [7,16].

In this study, we present the complete hepatic metabolism pathway of nebivolol established according to HLM assay with the use of LC-HR-MS coupled with chemometric method. Moreover, toxicity of the new metabolite (2-amino-1-(6-fluoro-3,4-dihydro-2*H*-1-benzopyran-2-yl)ethan-1-ol) was evaluated with the use of several in silico models.

## 2. Results and Discussion

### 2.1. HLM Biotransformation of Nebivolol

**Figure 1:**
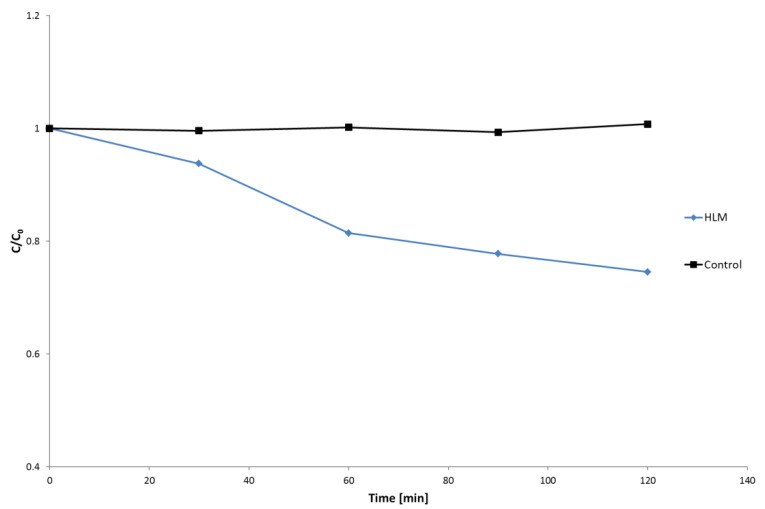
Biotransformation of nebivolol in HLM.

The in vitro biotransformation kinetics study of nebivolol in HLM was performed based on the evaluation of abundance of the parent ion (*m*/*z* 406.1824), in the studied time, the range of incubation (0–120 min) and moderate metabolism of the analyzed drug were observed (Figure 1). The obtained results showed that during 60 min of hepatic microsomes incubation about 20% of nebivolol was metabolized, and then the reactions of biotransformation slowed down. Taking this into account, the time of 60 min of HLM incubation with nebivolol was selected to perform all chemometric and qualitative analysis.

### 2.2. Chemometric Analysis

**Figure 2:**
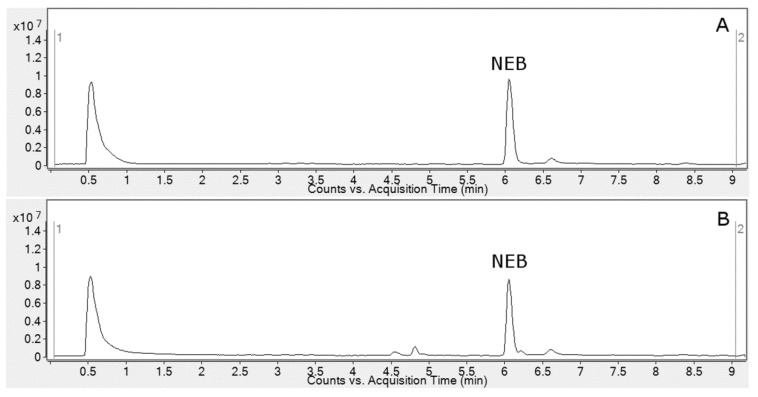
Total ion current chromatograms (TIC) of nebivolol (NEB) after 60 min incubation with HLM ((**A**)—control; (**B**)—sample).

The reversed-phase ultra-high pressure liquid chromatography (UHPLC) system coupled with high-resolution mass spectrometry (HR-MS) was utilized for the registration of HLM metabolic profiles of nebivolol. As shown in Figure 2, the registered profiles are quite similar and differences between control and test samples were observed only in the retention time in the range of 4.5–4.9 min. Taking this into account, in order to obtain accurate metabolite profiling of the recorded chromatograms, multivariate chemometric analysis was applied.

**Figure 3:**
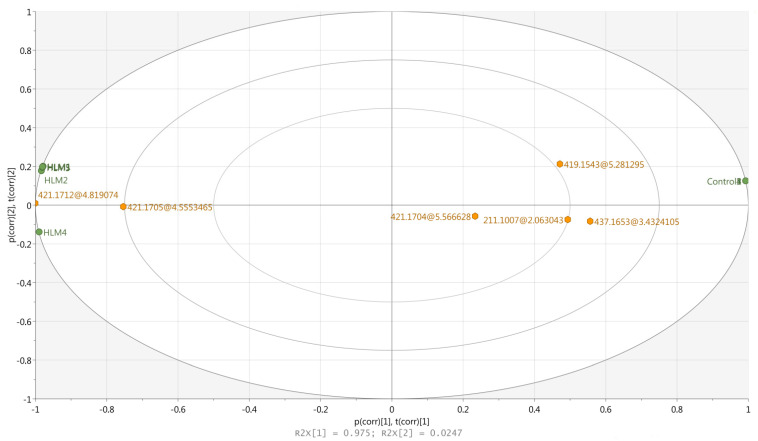
PCA biplot of HLM and Control samples (green) with the loadings (orange) of the entities (ions representing of the potential metabolites, expressed as nominal masses in Da).

In order to carry out the chemometric analysis all the obtained metabolite profiles (10 chromatograms) were aligned with the MPP software giving 39 entities (ions with the specific retention times, charge, and *m*/*z* value). After a build-in MPP filtration including sample abundance and moderated t-test (*p* < 0.05, FC ≥ 2), six entities (ions presented in Table 1) were finally selected for the chemometric study (input data matrix). Comparison of the obtained statistical data expressed as the box-whisker plots are presented in Figure S6. It can be observed that all the obtained results are characterized by very low dispersion of the data and only in the case of the **M4** metabolite was significantly higher skewness registered. Based on this, data principal component analysis (PCA) was performed using SIMCA software and the first two principal components (PC) explained 99.9% of the total variance (R^2^ = 0.975 − PC1; R^2^ = 0.024 − PC2). As shown in Figure 3, the negative control samples stood out from the HLM profiles, which clearly confirms the occurrence of the specific metabolic reactions. Additionally, it can be also observed that the ions representing potential metabolites were categorized in two groups—those placed closer to the HLM samples (**M1** and **M2**), and those located between the HLM and the control group (**M3**–**M6**). The first group represented the most abundant ions (region of 4.5–4.9 min on the chromatogram—Figure 2B) characteristic for the main and well-known hydroxylated (+16 Da) metabolites of nebivolol. The second group of the analyzed entities represented the moderate and lower abundance ions, which can also be found as a well-described oxidative metabolites (+16 Da, +14 Da, +32 Da) of the parent compound. It should be noticed that in this group, one ion with unique low mass—211.1007 Da (*m*/*z* 212.1083), not reported in the literature data so far, was also found. Taking that into account, this ion as well as the rest of the chemometrically selected entities were subsequently submitted to HR-MS/MS analysis, which allows much more accurate structural elucidation of the analyzed compounds than the low-resolution MS technique. 

### 2.3. Metabolite Identification

**Figure 4:**
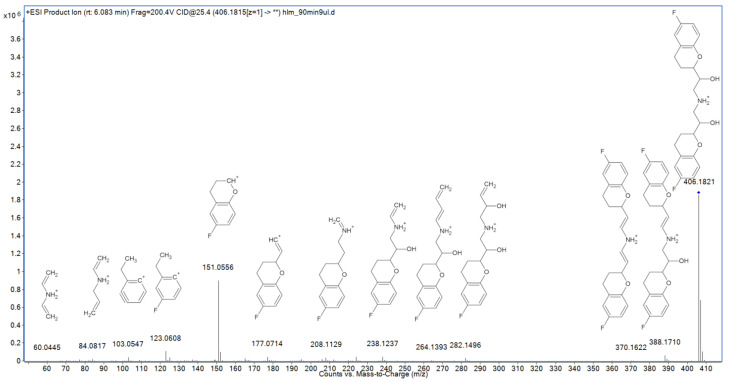
MS/MS spectrum and fragmentation pattern of nebivolol.

MS/MS fragmentation of nebivolol may result in elimination of two hydroxyl groups (as water molecules) and formation of the *m*/*z* 388.1710 and 370.1622 ions (Figure 4). Another fragmentation path includes elimination of a 6-fluoro-3,4-dihydro-2*H*-1-benzopyran (6-FDBP) fragment (*m*/*z* 151.0556 ion) and formation of the *m*/*z* 238.1237 ion, which undergoes further decomposition to *m*/*z* 224.1038, 208.1129, 195.0825, 177.0714, and 165.0711 ions (however, some of them may also have origins in the preliminarily dehydroxylated fragments). Subsequent fragmentation of 6-FDBP leads to formation of several low-mass ions such as *m*/*z* 137.0402, 123.0608, and 103.0547. Third probable fragmentation path includes direct decomposition of 6-FDBP, resulting in the formation of *m*/*z* 282.1496 and 264.1393 ions, as well as two low-mass fragments, representing secondary amine derivatives: *m*/*z* 84.0817 and 70.0663.

**Figure 5:**
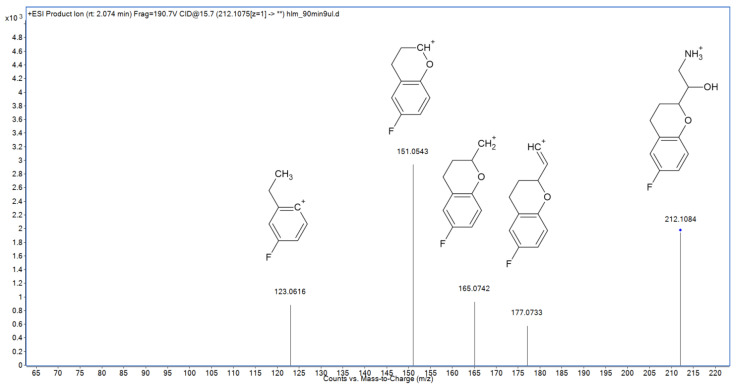
MS/MS spectrum and fragmentation pattern of novel nebivolol metabolite (**M6**).

In the following study, six hepatic metabolites of nebivolol were found and identified using high-resolution mass spectrometry (Table 1). Five of them are previously reported products of alicyclic and aromatic hydroxylation, alicyclic dihydroxylation, and alicyclic oxidation [15]. Their fragmentation MS/MS spectra are presented in the Supplementary Material (Figures S1–S5). However, the sixth metabolite—identified as a product of nebivolol N-dealkylation—was not described so far. Its MS/MS spectrum was shown in Figure 5. The major fragmentation ion—*m*/*z* 151.0543—is typical also for the parent compound, and represents 6-FDBP, which undergoes further decomposition forming the *m*/*z* 123.0616 ion. Two remaining fragmentation ions were also observed in the MS/MS spectrum of nebivolol—*m*/*z* 177.0733, which corresponds to elimination of both ammonia and water molecules followed by its subsequent degradation leading to formation of the *m*/*z* 165.0742 ion. On the other hand, ions representing secondary amine fragments such as *m*/*z* 84.0817 and 70.0663 were not detected in the spectrum, which confirms that N-dealkylation indeed took place in the case of **M6**.

### 2.4. Hepatic Biotransformation Pathways

**Figure 6:**
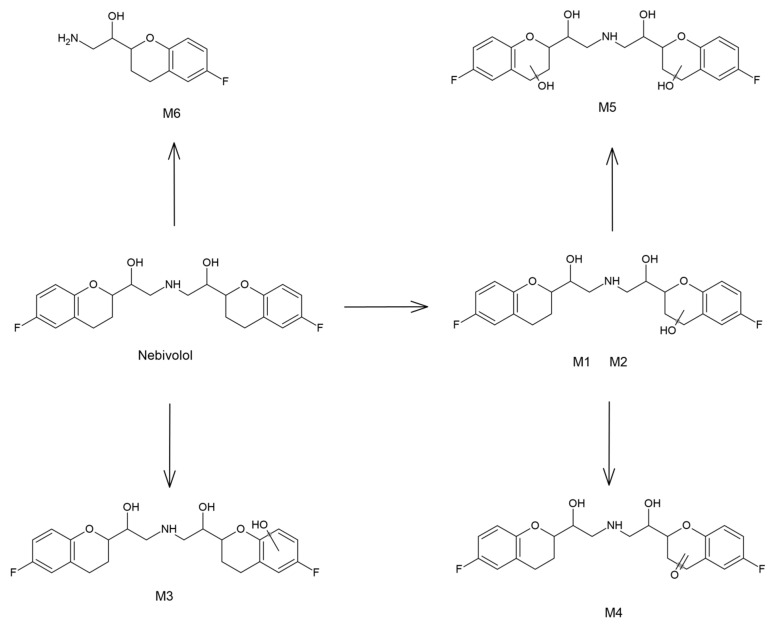
The proposed hepatic metabolic pathway of nebivolol.

Considering that HLM procedure was applied in this study, the identified biotransformation reactions can be used to propose the hepatic phase I metabolism pathway of nebivolol, which has not been described so far (Figure 6). The major metabolic path includes alicyclic hydroxylation of 6-FDBP, leading to formation of **M1** and **M2**—two hydroxy metabolites, presumably 3- and 4-hydroxynebivolol. Subsequent alicyclic hydroxylation, which takes place in the second 6-FDBP gives **M5**—another product of biotransformation. Noteworthy, only one dihydroxynebivolol derivative was detected and identified, which indicates that only one of the monohydroxy metabolites (**M1** or **M2**) is susceptible to further hydroxylation. A similar situation was observed in the case of **M4**, which was identified as a product of alicyclic oxidation of the parent compound. This metabolite was probably formed as a consequence of hydroxynebivolol dehydrogenation; however, only one compound of this nature was found, which indicates that only **M1** or **M2** can undergo this type of metabolic reaction. **M3** was identified as another hydroxylation product; however, in this case the reaction probably took place in the aromatic ring of 6-FDBP. The last metabolic reaction has not been described so far and involves N-dealkylation of nebivolol, which leads to the formation of a primary amine metabolite—previously unreported in the literature.

Interestingly, many of the known nebivolol metabolites were not detected in this study. Nevertheless, it should be noted that other papers discussing this topic involved studies on different biological matrices, such as urine [15]. The absence of biotransformation products, such as alicyclic-hydroxy-keto-nebivolol, alicyclic-aromatic-dihydroxynebivolol, or products of the oxidative N-dealkylation, indicates that these metabolites are not formed in liver but in the other tissues of the human organism.

### 2.5. In Silico Assessment of Toxicity

In order to preliminarily compare toxicological properties of the novel metabolite of nebivolol (**M6**) with the parent compound and the already known biotransformation products, the computational toxicity evaluation methods were applied in this study. Acute toxicity to rodents, mutagenicity, and developmental toxicity endpoints were chosen and calculated using ACD/Labs Percepta (toxicity to rodents and mutagenicity) and T.E.S.T. (toxicity to rats, mutagenicity, and developmental toxicity) software.

The results concerning acute toxicity to rodents were presented in Table 2. The obtained LD50 values (expressed as log _mg/kg_) indicate that a new metabolite may exhibit lower toxicity towards mice (with the exception of subcutaneous administration) than nebivolol. In the case of rats, opposite results are observed—**M6** should be considered more toxic than the parent compound when administered orally. Noteworthy, both applied models showed the same prediction trend; however, T.E.S.T. software estimated considerably lower toxicity of the parent compound than Percepta. On the other hand, the new metabolite exhibits significantly lower toxic properties than nebivolol after intraperitoneal administration. Other metabolites generally possess properties closer to nebivolol. The distinctive exception is the Mouse OR Percepta model, which predicted that the parent compound should be the most toxic among all the studied compounds.

Interestingly, the mutagenicity predictions (expressed as the probability of the positive Ames test outcome) clearly indicate that the novel metabolite possesses explicitly higher toxic properties than nebivolol (Table 2). According to both applied models, the parent compound exhibits negligible mutagenic potential—even below 0.1 in the case of the Percepta prediction. Conversely, mutagenicity of **M6** cannot be definitely excluded, especially according to the Percepta model, which predicted over 0.5 probability of the positive Ames test. Interestingly, mutagenicity predictions for the other metabolites are highly consistent according to the Percepta software—all of the known biotransformation products possess low toxic potential, similar to the parent compound. On the other hand, T.E.S.T predictions are much more divergent. Some of the metabolites possess relatively low mutagenic potential (**M2** and **M3**), while other may be more harmful than **M6** (**M1** and **M4**).

Developmental toxicity results, obtained using solely T.E.S.T. software, indicate that all studied compounds may exhibit toxic properties, but are more pronounced in the case of several metabolites, including **M6** (Table 2).

## 3. Materials and Methods

### 3.1. Chemicals and Reagents

Nebivolol hydrochloride (1-(6-fluoro-3,4-dihydro-2*H*-chromen-2-yl)-2-[[2-(6-fluoro-3,4-dihydro-2*H*-chromen-2-yl)-2-hydroxyethyl]amino]ethanolhydrochloride) was obtained from commercially available pharmaceutical formulation—Nebilet 5 mg tablets (Berlin-Chemie Menarini, Glienicker Weg, Germany) containing racemic mixture of SRRR-nebivolol and RSSS-nebivolol (1:1). Twenty tablets (equivalent of 40.5 mg of nebivolol) were grounded in mortar, then weighted and swept for 5 min with 25 mL of methanol, and then diluted with water to the working concentration. 

Water (LC-MS grade), β-nicotinamide adenine dinucleotide 2′-phosphate reduced tetrasodium salt hydrate (NADPH), HLM, sodium phosphate monobasic monohydrate salt and sodium phosphate dibasic anhydrous salt were obtained from Sigma-Aldrich (St. Louis, CA, USA). Acetonitrile (LC-MS grade) was purchased from Merck (Darmstadt, Germany) and 98% formic acid (MS grade) was obtained from Fluka (Taufkirchen, Germany). 

### 3.2. In Vitro Simulation of Metabolism by HLM

Metabolism study was performed in vitro with the use of human liver microsomes (HLM) fraction [17]. Incubation system consisted of 40 μM substrate, 55 mM phosphate buffer (pH 7.4), and 0.5 mg mL^−1^ HLM. Following 2 min pre-incubation period at 37 °C, the metabolic reactions were initiated by addition of 10 μL NADPH (20 mM). The reaction was terminated after 0, 30, 60, 90, and 120 min of incubation with the use of ice-cold acetonitrile–methanol mixture (1:1). Precipitated samples were centrifuged at 15,000 rpm for 10 min at 4 °C, and the supernatants (40 μL) were transferred into the vials for LC-HR-MS analysis. The negative control samples were prepared with the same manner without addition of NADPH solution. 

### 3.3. Analytical Procedures

The LC-HR-MS analysis was performed with the use of Agilent high-resolution Q-TOF system series 6520 and UHPLC system series 1290 (Agilent Technologies, Santa Clara, CA, USA) with Kinetex C18 (2.1 × 50 mm, dp = 1.7 μm) column and C18 precolumn guard (Phenomenex, Torrance, CA, USA). In order to perform both qualitative and quantitative analysis of the studied processes, the MS detector was tuned in a positive mode in extended dynamic range (2 GHz). MassHunter workstation software in version B.04.00 (Agilent Technologies, Santa Clara, CA, USA) was used for the control of the system, data acquisition, qualitative, and quantitative analysis. To ensure accuracy of the masses’ measurements, a reference mass correction was applied and masses 121.050873 and 922.009798 were used as lock masses. MS detection based on the extracted ion current chromatograms (EIC) was applied for the quantitative analysis of nebivolol, and then auto MS/MS mode (using abundance algorithm) was used for registration of their fragmentation spectra. All the chromatographic and spectrometric parameters are described in Table S1.

### 3.4. Chemometric and Toxicity Prediction Software

Chemometric analysis (PCA) was performed with the use of Mass Profiler Professional (MPP) software version 12.61 (Agilent and Strand Life Sciences Pvt. Ltd. Santa Clara, CA, USA) and SIMCA software version 16.0.2 (Umetrics, MKS Instruments Inc., Goettingen, Germany). Before the analysis, raw data profiles were pre-treated using molecular feature extraction (MFE) algorithm from the Mass Hunter Qualitative Analysis software version B.06.00 (Agilent Technologies, Santa Clara, CA, USA). 

In silico toxicity including mutagenicity (expressed as a probability of positive outcome of the Ames test) were evaluated using Toxicity Estimation Software Tool (T.E.S.T.) v. 4.2.1 (United States Environmental Protection Agency, Washington, DC, USA), ACD/Percepta 14.0.0 (ACD/Labs, 2015 Release, Advanced Chemistry Development, Inc., Toronto, ON, Canada) software.

## 4. Conclusions

In this study, a complete phase I hepatic metabolism pathway of a cardiovascular drug nebivolol was proposed. With the use of LC-HR-MS coupled with the multivariate chemometric method, a new biotransformation product of nebivolol (2-amino-1-(6-fluoro-3,4-dihydro-2*H*-1-benzopyran-2-yl)ethan-1-ol) was identified and characterized. In contrast to the main metabolites, described as an effect of the hydroxylation reaction, the novel compound was found to be a product of N-dealkylation. 

The in silico analysis of toxicity showed that, compared to the parent compound, the identified new metabolite (**M6**) generally possesses lower toxicity towards mice and higher towards rats. Especially interesting are outcomes of the mutagenicity estimations, which indicate a considerably higher mutagenic potential of this metabolite, which may require further investigational follow-up.

Taking the above into account, the development of the new analytical methods for the determination of nebivolol and its metabolites, including the new one, in biological materials should be also considered. 

## Figures and Tables

**Table 1:** Q-TOF accurate mass elemental composition of the analyzed metabolites.

Name	Reaction Type	Retention Time (min)	Measured Mass (*m*/*z*)	Theoretical Mass (*m*/*z*)	Mass Error (ppm)	Molecular Formula [M + H]^+^
NEB	–	6.05	406.1831	406.1824	1.72	C_22_H_26_F_2_NO_4_
**M1**	Al-OH	4.84	422.1761	422.1774	3.07	C_22_H_26_F_2_NO_5_
**M2**	Al-OH	4.57	422.1785	422.1774	2.61	C_22_H_26_F_2_NO_5_
**M3**	Ar-OH	5.58	422.1761	422.1774	3.08	C_22_H_26_F_2_NO_5_
**M4**	Al-OH, Deh	5.30	420.1618	420.1617	0.24	C_22_H_24_F_2_NO_5_
**M5**	Al-OH	3.45	438.1726	438.1723	0.68	C_22_H_26_F_2_NO_6_
**M6**	N-dealk	2.08	212.1083	212.1081	0.94	C_11_H_15_FNO_2_

Al-OH—alicyclic hydroxylation; Ar-OH—aromatic hydroxylation; Deh—dehydrogenation; N-dealk—N-dealkylation.

**Table 2:** Toxicity of nebivolol and **M1**–**M6** metabolites.

Toxicity Model	NEB	M6	M1	M2	M3	M4	M5
Mouse IP	2.34	2.88	2.45	2.42	2.4	2.36	2.45
Mouse OR	2.59	2.68	2.68	2.65	2.67	2.81	2.87
Mouse IV	1.72	2.04	1.73	1.83	1.78	1.8	1.75
Mouse SC	3.32	2.84	3.45	3.38	3.36	3.3	3.53
Rat IP	1.70	2.54	1.76	1.85	1.84	1.93	1.81
Rat OR	2.83	2.76	2.88	2.86	3.01	2.8	2.96
Rat OR T.E.S.T.	3.15	2.64	3.06	3.4	2.67	3.01	2.77
Ames Percepta	0.06	0.61	0.08	0.06	0.05	0.07	0.08
Ames T.E.S.T.	0.10	0.45	0.46	0.21	0.5	0.56	0.46
Developmental Toxicity	0.62	0.76	0.71	0.57	0.81	0.75	0.65

IP—intraperitoneal; OR—oral; IV—intravenous; SC—subcutaneous; rodent toxicity (LD_50_) expressed as log _mg/kg_; mutagenicity and developmental toxicity expressed as probability of the toxic effect (0—no effect; 1—certain effect).

## Data Availability

Data is contained within the article or Supplementary Materials.

## Supplementary Materials

The following are available online, Table S1: Applied LC and MS parameter; Figure S1: MS/MS spectrum and fragmentation pattern of **M1** metabolite; Figure S2; MS/MS spectrum and fragmentation pattern of **M2** metabolite; Figure S3: MS/MS spectrum and fragmentation pattern of **M3** metabolite; Figure S4: MS/MS spectrum and fragmentation pattern of **M4** metabolite; Figure S5: MS/MS spectrum and fragmentation pattern of **M5** metabolite; Figure S6: Box-whisker plots (normalized intensity values) for the entities (loadings) used in PCA analysis. (The plots shows the median in the middle of the box, the 25th percentile and the 75th percentile. The lowest datum is within 1.5 IQR (Interquartile range) of the lower quartile, and the highest datum is within 1.5 IQR of the upper quartile.).

## Sample Availability

Samples of the compounds are not available.
