# Supplementary material for: Identification of the New Metabolite of Nebivolol Using Liquid Chromatography Coupled with High-Resolution Mass Spectrometry and Chemometrics
Source: Molecules. 2022 Jan 24;27(3):763. doi: 10.3390/molecules27030763 (PMC8839339; doi:10.3390/molecules27030763)
Supplement: Supplementary file 1 [file molecules-27-00763-s001.zip › molecules-1544381-supplementary.pdf]

**Supplementary material**

# Identification of the New Metabolite of Nebivolol using Liquid Chromatography Coupled with High-Resolution Mass Spectrometry and Chemometrics

Jakub Trawiński, Michał Wroński, Maciej Gawlik and Robert Skibiński*

Department of Medicinal Chemistry, Faculty of Pharmacy, Medical University of Lublin, Jaczewskiego 4, 20-090 Lublin, Poland

* Corresponding author. Tel.: +48 81 4487390; fax: +48 81 4487380.

E-mail address: robert.skibinski@umlub.pl

Table S1. Applied LC and MS parameter

| Device | Parameter | Value |
|---|---|---|
| LC | Solvents | A – 0.1% aqueous solution of HCOOH |
| | | B – acetonitrile |
| | Gradient | 10% B to 50% B |
| | Analysis time | 9 min |
| | Post-time equilibration | 2 min |
| | Flow rate | 0.3 mL min$^{-1}$ |
| | Injection volume | 3 µL |
| | Column temperature | 35 °C |
| MS | Ion source | Electrospray (ESI) |
| | Mode | Positive |
| | Source temperature | 325 °C |
| | Drying gas flow | 10 L min$^{-1}$ |
| | Nebulizer pressure | 40 psig |
| | Capillary voltage | 3500 V |
| | Fragmentor voltage | 175 V |
| | Skimmer voltage | 65 V |
| | Octopole voltage | 750 V |
| | Mass range | 90 – 950 *m/z* |
| | Acquisition rate | 1.5 spectra s$^{-1}$ |

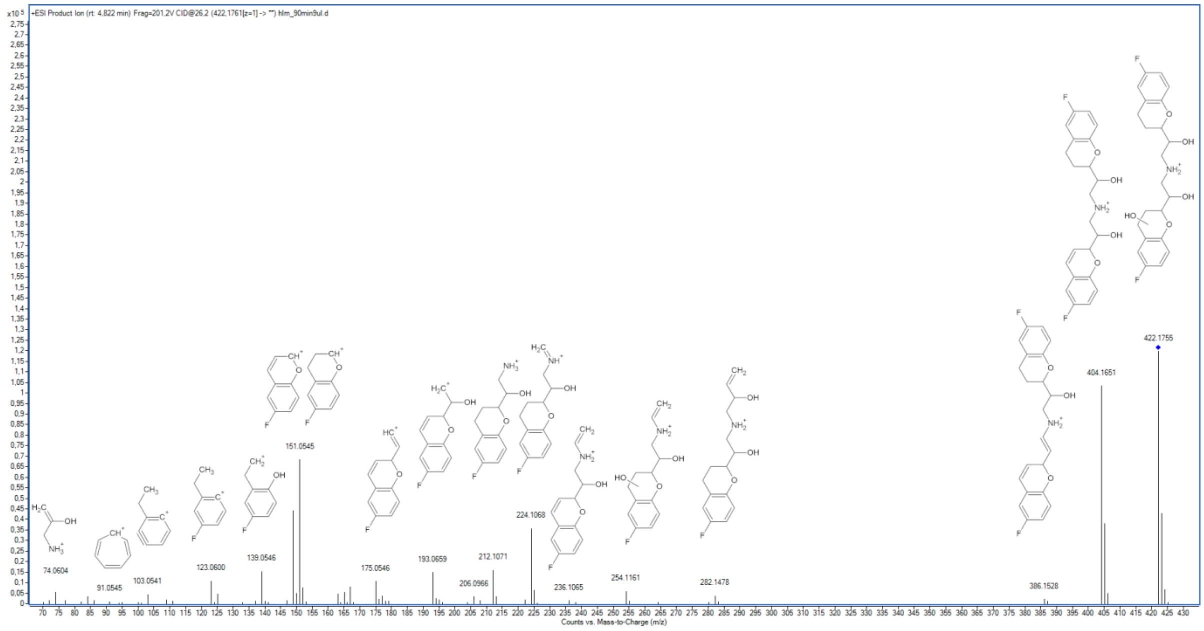


Figure S1. MS/MS spectrum and fragmentation pattern of M1 metabolite

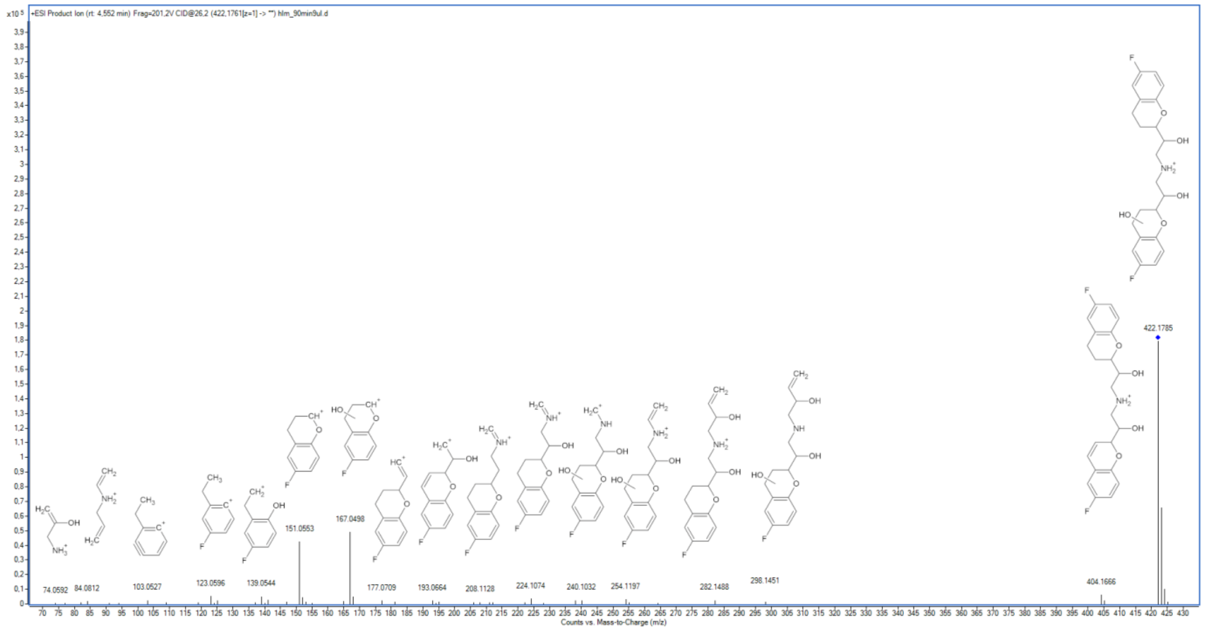

Figure S2. MS/MS spectrum and fragmentation pattern of M2 metabolite

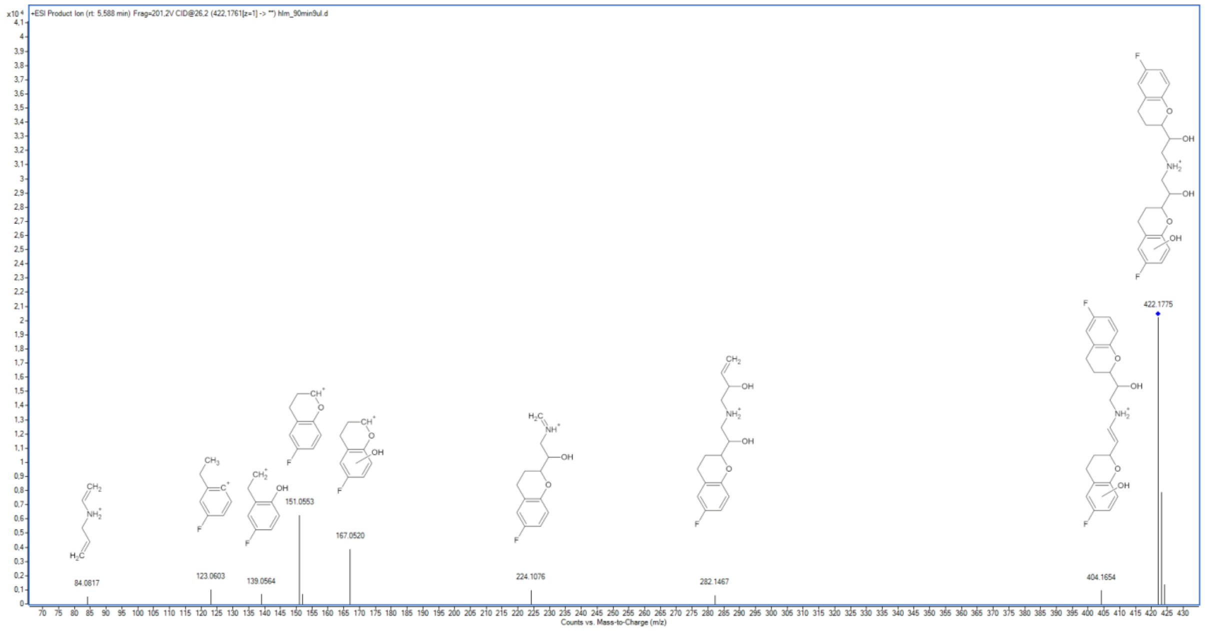

Figure S3. MS/MS spectrum and fragmentation pattern of M3 metabolite

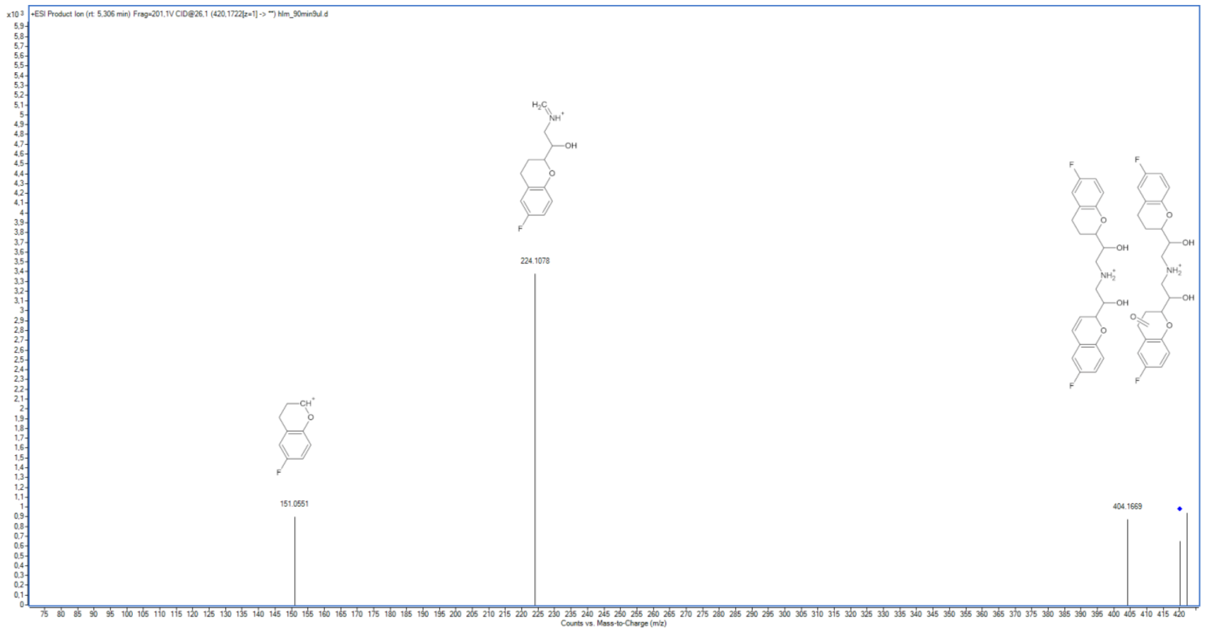


Figure S4. MS/MS spectrum and fragmentation pattern of M4 metabolite

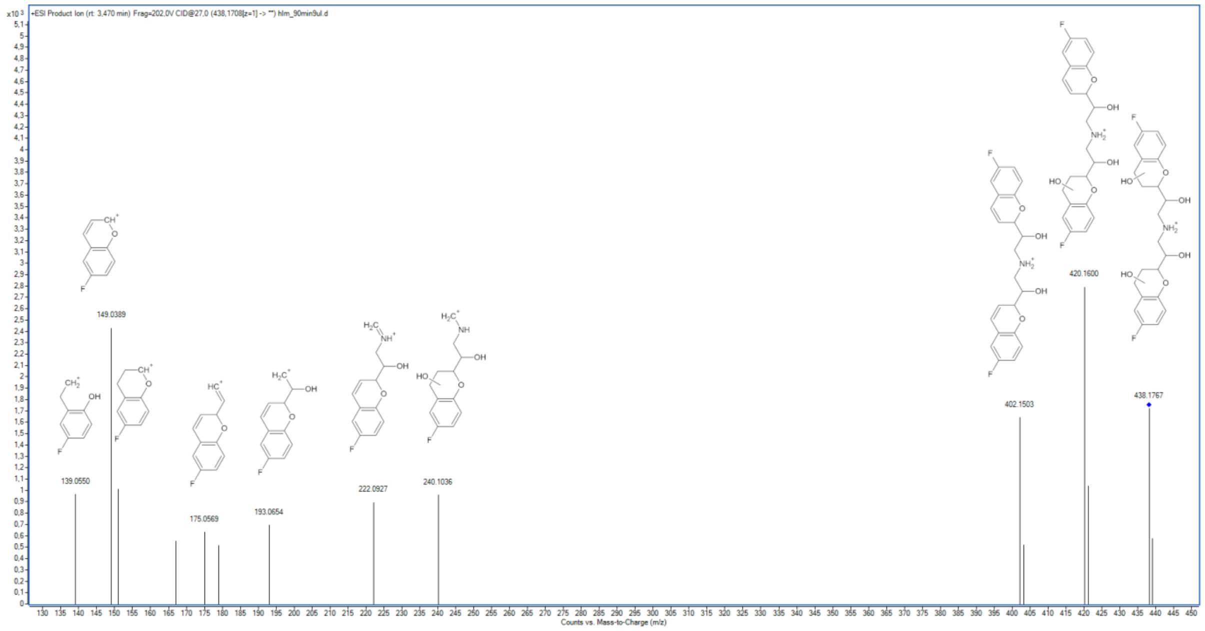


Figure S5. MS/MS spectrum and fragmentation pattern of M5 metabolite

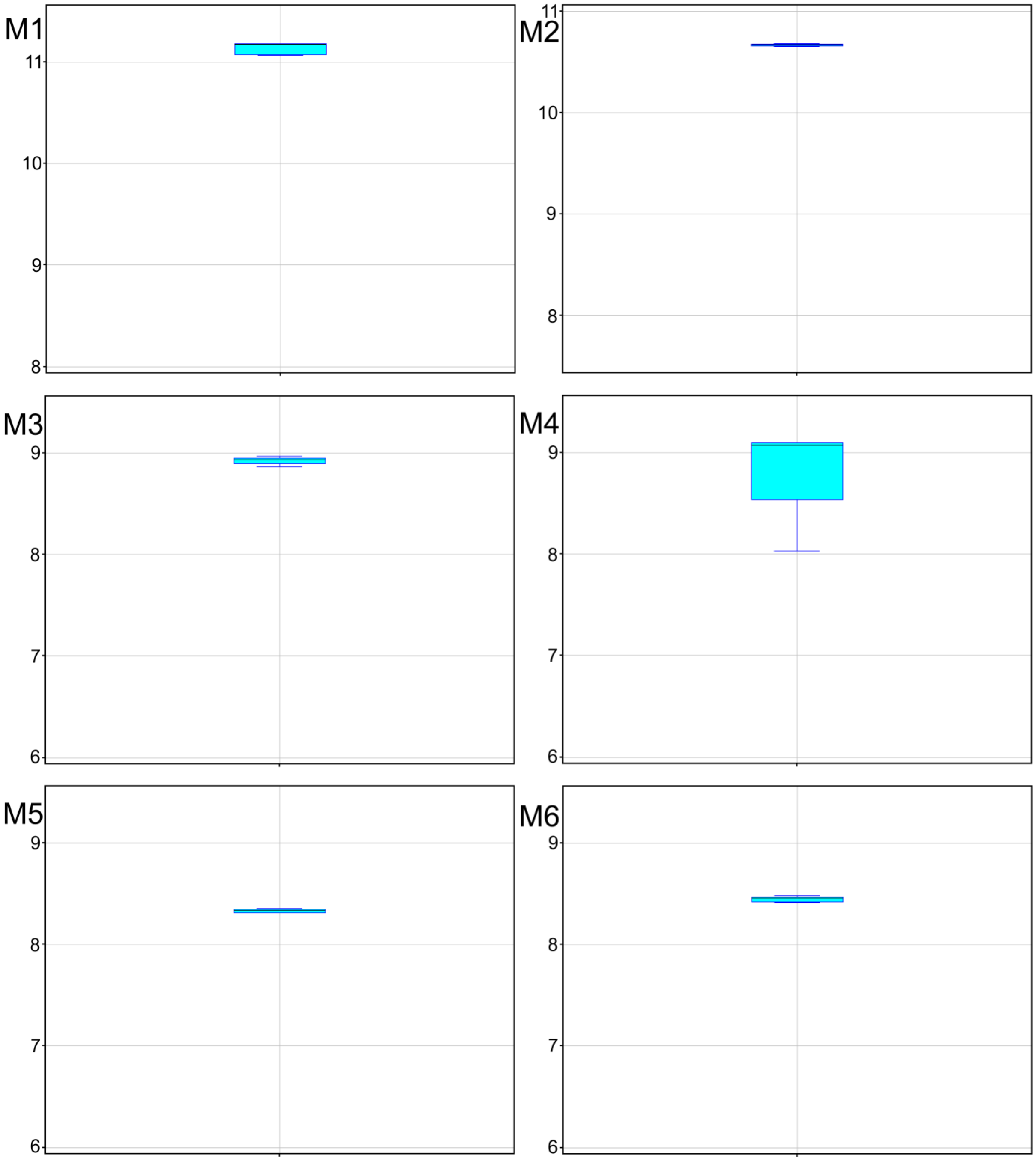


Figure S6. Box-whisker plots (normalized intensity values) for the entities (loadings) used in PCA analysis. (The plots shows the median in the middle of the box, the 25th percentile and the 75th percentile. The lowest datum is within 1.5 IQR (Interquartile range) of the lower quartile, and the highest datum is within 1.5 IQR of the upper quartile.)
